# Using a Neural Network Architecture for the Prediction of Neurologic Outcome for Out-of-Hospital Cardiac Arrests Using Hospital Level Variables and Novel Physiologic Markers

**DOI:** 10.3390/bioengineering12020124

**Published:** 2025-01-29

**Authors:** Martha Razo, Pavitra Kotini, Jing Li, Shaveta Khosla, Irina A. Buhimschi, Terry Vanden Hoek, Marina Del Rios, Houshang Darabi

**Affiliations:** 1 Department of Mechanical and Industrial Engineering, University of Illinois Chicago, 942 W Taylor St., Chicago, IL 60607, USA; mrazo3@uic.edu; 2 Department of Emergency Medicine, University of Illinois Chicago College of Medicine, Chicago, IL 60612, USA; pkotini@uic.edu (P.K.); jingli92@uic.edu (J.L.); skhosl2@uic.edu (S.K.); tvh@uic.edu (T.V.H.); 3 Department of Obstetrics and Gynecology, University of Illinois Chicago College of Medicine, Chicago, IL 60612, USA; irina@uic.edu; 4 Department of Medicine, University of Chicago Biological Sciences Division, Chicago, IL 60637, USA; marina.delrios@bsd.uchicago.edu

**Keywords:** neural network, prediction model, SHAP analysis, cardiac arrest

## Abstract

Out-of-hospital cardiac arrest (OHCA) is a major public health burden due to its high mortality rate, sudden nature, and long-term impact on survivors. Consequently, there is a crucial need to create prediction models to better understand patient trajectories and assist clinicians and families in making informed decisions. We studied 107 adult OHCA patients admitted at an academic Emergency Department (ED) from 2018–2023. Blood samples and ocular ultrasounds were acquired at 1, 6, and 24 h after return of spontaneous circulation (ROSC). Six classes of clinical and novel variables were used: (1) Vital signs after ROSC, (2) pre-hospital and ED data, (3) hospital admission data, (4) ocular ultrasound parameters, (5) plasma protein biomarkers and (6) sex steroid hormones. A base model was built using 1 h variables in classes 1–3, reasoning these are available in most EDs. Extending from the base model, we evaluated 26 distinct neural network models for prediction of neurological outcome by the cerebral performance category (CPC) score. The top-performing model consisted of all variables at 1 h resulting in an AUROC score of 0.946. We determined a parsimonious set of variables that optimally predicts CPC score. Our research emphasizes the added value of incorporating ocular ultrasound, plasma biomarkers, sex hormones in the development of more robust predictive models for neurological outcome after OHCA.

## 1. Introduction

Numerous endeavors have been made to create prediction models concerning the outcome of various medical conditions. Deep learning with Neural Networks (NNs) have shown an extraordinary performance in prediction across research in many industries including the medical field. Several significant disorders such as diabetes [1], coronavirus disease 2019 (COVID-19) [2], paralytic ileus (PI) [3] and heart failure (HF) [4] have been analyzed using deep learning models to predict outcomes. Yet, there remains a gap for one of the most lethal public health problems in the United States, out-of-hospital cardiac arrest (OHCA).

Globally, the incidence of OHCA ranges from 20 to 140 per 100,000 people, with survival rates from 2% to 11% across various regions [5]. Cardiac arrest claims more lives than colorectal cancer, breast cancer, prostate cancer, influenza, pneumonia, auto accidents, HIV, firearms, and house fires combined [6]. Cardiac arrest is defined as a sudden and complete cessation of effective cardiac contraction resulting in lack of circulation to the rest of the body and OHCA refers to the scenario when cardiac arrest occurs outside of the hospital and presents unique challenges related to availability of bystander cardiopulmonary resuscitation and distance from a major hospital among others [7]. The brain is the most sensitive to the loss of circulation and the deleterious cascade of ischemia-reperfusion injury among those who survive can lead to permanent brain injury or neurological dysfunction which results in significant mortality and disability [2]. As result, the most valuable outcome measure for OHCA is the neurological outcome.

Motivated by the successful implementation of deep learning models to predict the outcome of cardiac arrest, in this exploratory study we aimed to develop a NN model using in-hospital variables and novel physiologic markers to predict neurological outcome by Cerebral Performance Category (CPC) in adult OHCA cases.

The goal of this study is to provide insight into if unexplored novel variables may contribute significantly to prediction of CPC. Optic Nerve Sheath Diameter (ONSD) by ocular ultrasound has been shown to be a possible non-invasive neurological monitoring tool and may have utility in predicting outcomes in post-cardiac arrest patients [8,9]. Yet this modality has not been tested as part of a bundle with the various care elements for post-cardiac arrest patients. Similarly, sex differences have been overlooked in critical illness and in biomarkers [10]. Building on the prior work on sex hormones [11] and preliminary work on previously unexplored biomarkers, these rarely available variables were also incorporated to test their potential contribution to neurological prediction. A NN architecture will allow us to conduct SHapley Additive exPlanations (SHAP) and Random Forest variable importance analysis to better understand how different clinical variables influence the neurologic outcome in OHCA patients. Findings from this study may possibly assist in adjusting hospital level procedures and implementing essential interventions to tilt the outcome towards the preservation of patients’ lives with minimal neurological impairment.

## 2. Materials and Methods

### 2.1. Data Source and Inclusion Criteria

For this study we recruited adult (age > 18 year), witnessed or unwitnessed OHCA cases who survived and were admitted at an urban academic Emergency Department from May 2018 to February 2023. Patients who could not be consented for the study (through a legally authorized representative) were not eligible. The study was reviewed and approved by institutional review boards of University of Texas Houston (UTH) where the blood samples were collected and University of Illinois Chicago (UIC) where the analysis was conducted. Written consent was obtained within 72 h after first blood draw from eligible family members. Patient demographics, cardiac arrest characteristics, clinical data in the pre-hospital care and during hospital course was abstracted from medical charts and de-identified data shared with UIC. Our analysis focused on clinical, ultrasound and biochemical data collected at time points that would provide insight into the progression of ischemic-reperfusion brain injury within hours to days. Data collection points were at 1, 6, 24 and 48 h after ROSC. Patient deaths subsequent to the 1 h blood draw were not excluded.

### 2.2. Clinical Variables

The study coordinator abstracted clinical variables from the medical record for each enrolled patient. These included demographic information, time of cardiac arrest, initial rhythm, whether bystander CPR was performed, time of return of spontaneous circulation, medications administered during Advanced Cardiac Life Support (ACLS) protocol, arrival time to the emergency department, time cooling protocol was initiated, presenting neurologic function as measured by Glasgow Coma Scale (GCS), initial vital signs, initial non-study lab values that are drawn as part of standard care (including complete blood count (CBC), basic metabolic panel (BMP), magnesium level, cardiac markers, coagulation studies, venous or arterial blood gas results, lactate level), whether the patient is intubated), and ED disposition status (admitted to cardiology care unit (CCU), medical intensive care unit (MICU), cath lab results, etc.).

Our primary outcome was neurologic outcome as defined by the Cerebral Performance Category (CPC) Score at 72 h and at discharge as calculated by the study site principal investigator. A CPC score 1–2 shows favorable neurological results, meaning the patient has mild to moderate disability and is able to perform independent activities, while a CPC score of 3–5 represents unfavorable outcomes for OHCA patients, encompassing severe disability, coma/vegetative state, and death [12]. Discharge status (alive or deceased) and discharge location (to home, nursing home, rehabilitation hospital, etc.) was also collected from the chart.

### 2.3. Ocular Ultrasound

After ROSC, by convenience sampling, trained emergency physicians obtained bilateral optic nerve sheath diameter (ONSD) measurements by bedside point-of-care ultrasound, at 1, 6, and 24 h in the transverse, oblique, and longitudinal planes for each eye, at each time point [8,9]. Time points for blood collection after ROSC were within 1, 6, 24, and 48 h after ROSC. Blood was collected by venipuncture or via a pre-existing arterial or venous catheter into EDTA tubes (for plasma collection). The first sample was collected either on arrival to the ED or in the Intensive Care Unit if the patient was transferred from another facility. Blood tubes were immediately placed on ice and centrifuged at 2000× *g* for 10 min. within 1 h from collection after which plasma was aliquoted and stored at −80 °C.

Plasma protein biomarkers were measured using the 40-plex assay (Meso Scale Diagnostics, Rockville, MD, USA) that comprised of a cytokine panel (10 cytokines), a chemokine panel (10 chemokines), a proinflammatory panel (additional 10 cytokines), a vascular injury panel (4 protein biomarkers), and an angiogenesis panel (6 protein biomarkers).

In addition, (6) plasma sex steroid hormones (Estrone, Estradiol, Testosterone, and Progesterone) values obtained by Liquid Chromatography Mass Spectroscopy were used. The methods for the sex hormones have been previously published [11].

### 2.4. Variable Grouping

We examined six different sets of data to assess their predictive value for CPC score. Three sets of variables are currently available in any emergency room: (1) Vital signs, (2) Pre-hospital and ED data, and (3) Hospital admission data. We also explored a second set of exploratory research variables consisting of (4) ONSD measurements, (5) plasma protein biomarkers.

The selected variables are listed in Supplement in Appendix A with yellow variables representing standard of care data (typically available for any post-ROSC patient) and blue colored variables representing the novel physiological markers. The data was split into 70% for training and 30% for testing the models. An equitable division of class 0 (CPC score of 3–5) and class 1 (CPC score 1–2) was employed in both the training and test set, ensuring that comparable proportions of minority groups were included in each group. The training and test procedure was repeated 30 times to search for optimal hyperparameters for the NN using a K-fold cross-validation architecture on the training set to evaluate the impact of parameters of the NN.

### 2.5. 1, 6, and 24 h CPC Score Prediction

**Figure 1:**
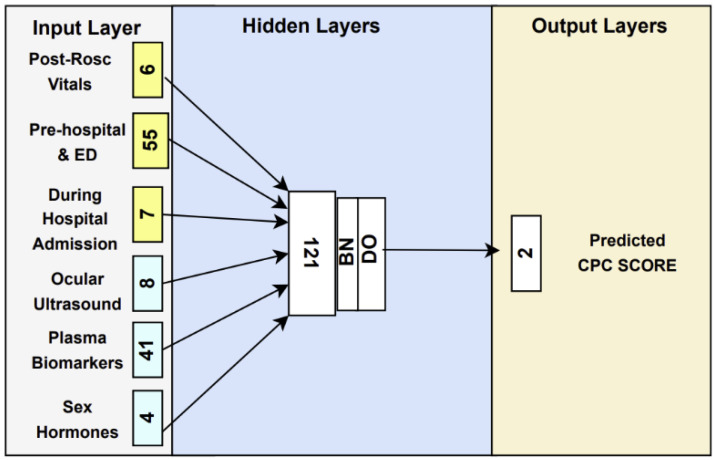
Architecture of Neural Network Model with Input Layer Variables.

The six datasets are inputted into a dense NN, and the outcome is predicted as either a CPC score of 1–2 or CPC of 3–5. The number of variables in each layer represents the number of neurons. Before the concatenation of the hidden layers, each input layer is individually connected to a separate hidden layer branch. These branches, along with the concatenated hidden layer, are then fed into two additional layers. All hidden layers utilize a Rectified Linear Unit activation function (ReLU), which have shown exceptional performance in the existing literature. Moreover, the branched first hidden layers are equipped with batch normalization (BN) layers to expedite convergence and enhance stability [13]. To introduce regularization, dropout (DO) layers with a rate of 5% are incorporated. A comprehensive depiction of this architecture can be found in Figure 1. The NN model was trained for 250 epochs with a batch size of 50. The output layer employed a softmax activation function to predict 1 h, 6 h and 24 h CPC score for patients with OHCA. The Adam optimizer function [14] was used with a learning rage of 4 × 10^−3^. To evaluate the proposed model, the AUROC method was utilized with DeLong’s procedure [15] for calculation of 95% CIs.

### 2.6. Baseline Model Development

**Figure 2:**
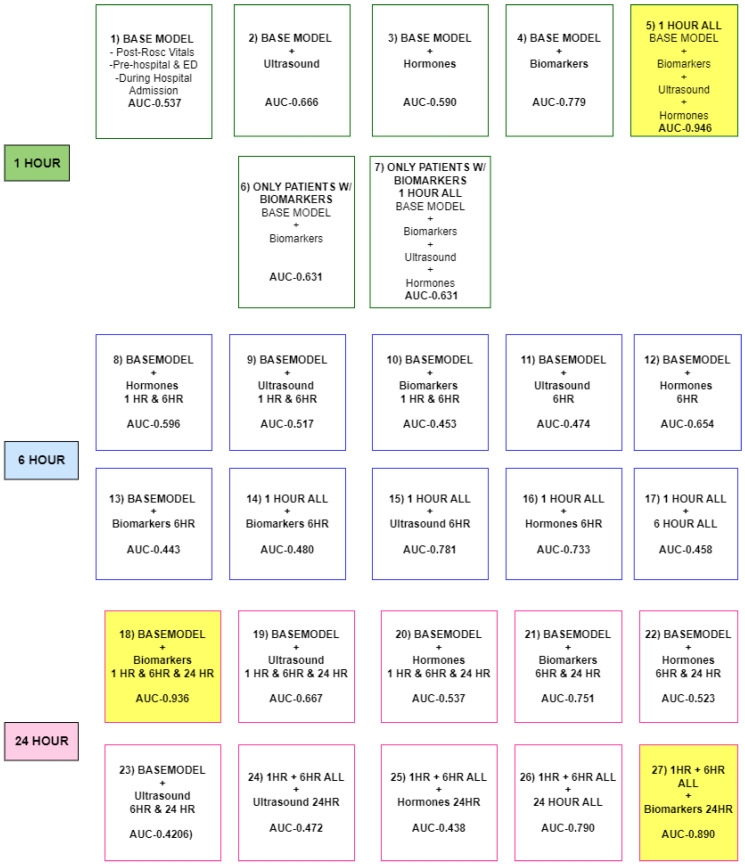
Summary of the results for the baseline model and proposed models.

**Figure A1:**
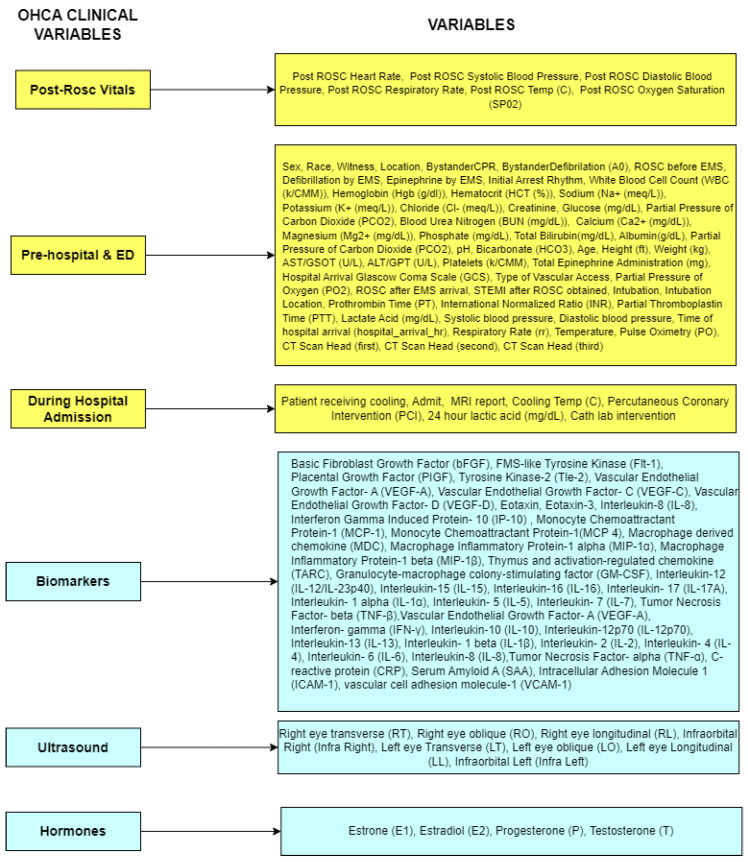
Dataset of variables used for the NN model for prediction of CPC score of OHCA patients.

In order to compare the results of the NN, a baseline model was built using variables that are readily available within one hour of ROSC (Figure A1). This baseline model is depicted as model 1 in Figure 2 and consists of the following categories of variables: (1) Vital signs after ROSC, (2) Pre-hospital and emergency department data, and (3) Hospital admission data. For models 2, 3, and 4 we tested different models using a combination of the research variables. For instance model 2 is the base model plus the ultrasound variables at 1 h. For model 5, we tested all datasets available at 1 h. For 1 h we noticed that the plasma biomarkers played an important role. Although plasma protein biomarker data was not available for every patient, given the significance of biomarkers in predicting the CPC score, models 6 and 7 were directed towards the subset of patients who possessed complete biomarker information, which amounted to 33 individuals.

### 2.7. Variable Impacts

To assess the influence of each variable on the predictions of our proposed model and determine which variable is specifically associated with CPC score, both Random Forest and SHAP analysis [16] was performed on the test set. SHAP values provide an explanation of the contribution of each feature to the prediction of an individual instance. This makes it possible to understand the impact of each feature on the prediction, and to identify which features are the most important drivers of the prediction. SHAP values are unique for each instance and are often visualized as bar plots or summary plots, allowing for easy interpretation. Using SHAP analysis, we were able to visually represent which variables have the greatest impact on the CPC score of OHCA. Similarly, Random Forest allows for the determination of the influence of each variable on the model prediction for an individual instance but providing a global approach [17].

### 2.8. Evaluation Metrics

The AUC score was selected as the evaluation metric because it is widely employed in the literature for comparing model performance [18] since AUC provides a comprehensive measure of a classification model’s ability to differentiate between classes at various decision thresholds. Additionally, AUC is recognized in the research community as the most suitable metric for assessing models when dealing with imbalanced class distributions [19]. The training cohort was used to develop the NN model and train the NN. The model with the highest AUC on the training set was considered the best model. This best model was then evaluated on the test cohort to assess its performance.

## 3. Results

A total of 107 patients with OHCA were studied. Of the total of 107 cases that were enrolled, 45% were female and 27% were White, 35% were discharged alive but only 20% of those alive had a CPC of 1–2.

### 3.1. Cohort Characteristics Model Completion

The description of the train and test cohorts is presented in Table 1. There were no significant differences between the cohorts in terms of outcome variable, demographics, or clinical variables. Out of 74 patients in the training cohort, 15 of them had a CPC score of 1–2 and 59 had a CPC score of 3–5. In the test cohort, 6 patients had a CPC score of 1–2 and 27 of them had a CPC score of 3–5. There was no significant difference in age between the training cohort and the test cohort (53 vs. 52 years, *p* = 0.804). Regarding gender, the training cohort contained 50% female and 50% male. The test cohort contained 33.3% female and 66.7% male. The distribution of sex was not significantly different between the cohorts [*p* = 0.838] as presented in Table 1. The proportions of white patients in the training and test cohorts were 27.0%, and 21.2% respectively. Similarly, for both clinical data, Bystander CPR [*p* = 0.339] and Initial Arrest rhythm [*p* = 0.937], there were not significantly different between cohorts.

### 3.2. Proposed and Baseline Models Performance

The results for the different proposed models are summarized in Figure 2 with the base model considering only the clinical and demographic data. The training split of the dataset was utilized for model selection. Figure 2 displays the AUC score for the train split of the data. The Wilcoxon test was applied to test whether the confidence intervals for all models were statistically different. The level of significance was selected to be 0.05. All the *p*-values were less than 0.05 for all models proving to be statistically different. The highlighted yellow boxes represent the higher performing models in terms of AUC score. Model 5, referenced as 1 HOUR ALL which includes the base model, ultrasound, sex hormones and plasma biomarkers at 1 h resulted in an AUC score of 0.946. Models utilizing data collected at 6 h were not high performing models. At 24 h, there are two models with better predictive performance. Model 27 includes the base model plus the biomarkers at only 24 h with an AUC score of 0.890. The highest performing model at 24 h is Model 18 which includes the base model, plus the biomarkers at all three time frames, 1, 6, and 24 h, resulting in an AUC of 0.936. The lowest performing models in terms of AUC score appear to be at hour 6. The poorest prediction model contained all the data for 1 h and 6 h plus only the hormones at 24 h with an AUC score of 0.438. The test results are listed in Table 2 below. Model 5 with the highest test AUC score of 0.924, followed by Model 18 with test AUC score of 0.898, and lastly Model 27 with a test AUC score of 0.886.

### 3.3. Random Forest Important Variables

**Figure 3:**
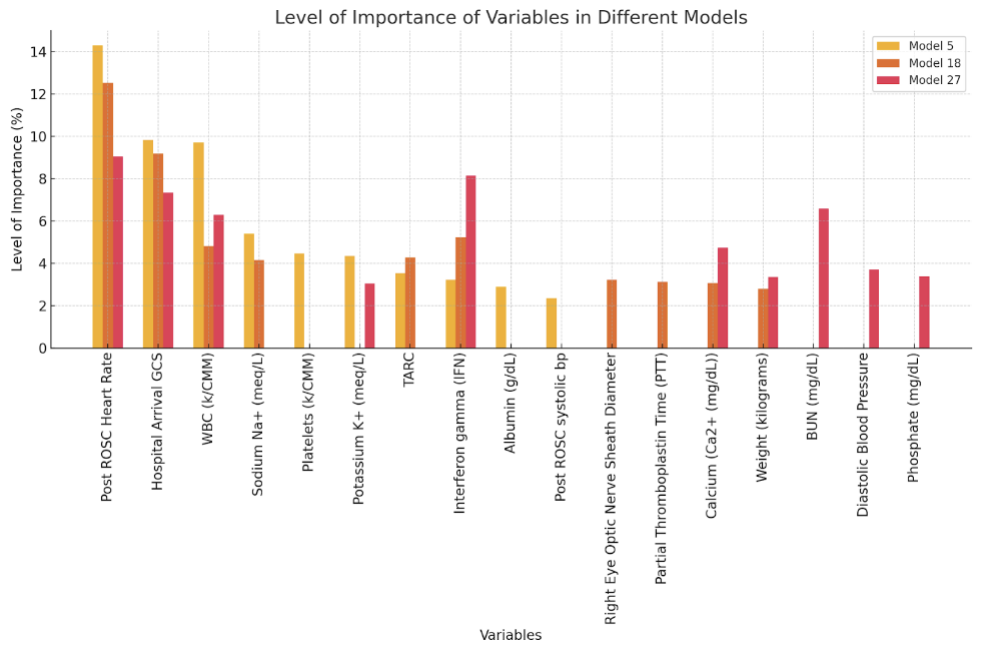
List of important variables in descending order for model 5, model 18, and model 27 of the training set.

For the three highlighted models with the highest AUC, the variable importance was captured. The top 10 most important variables contributing to the outcome of CPC score are summarized in Figure 3 below. For all three models, Post ROSC Heart Rate was considered to be the most important variable contributing to the CPC score when inputting all the 121 variables for the first hour. Post ROSC Heart Rate contributed power to the CPC score accounting for 14.29% towards the prediction. While the Post ROSC systolic blood pressure variable had the least contribution for the model with 1 HOUR ALL, contributing 2.36% towards CPC outcome prediction.

### 3.4. SHAP Value Analysis

Random Forest has the capability to capture the overall impact of each feature, but it lacks the ability to provide specific information regarding the local effect of individual variables. To address this limitation, we employed SHAP analysis, which allows us to gain a localized understanding of the contribution made by each variable towards the prediction of CPC performance.

**Figure 4:**
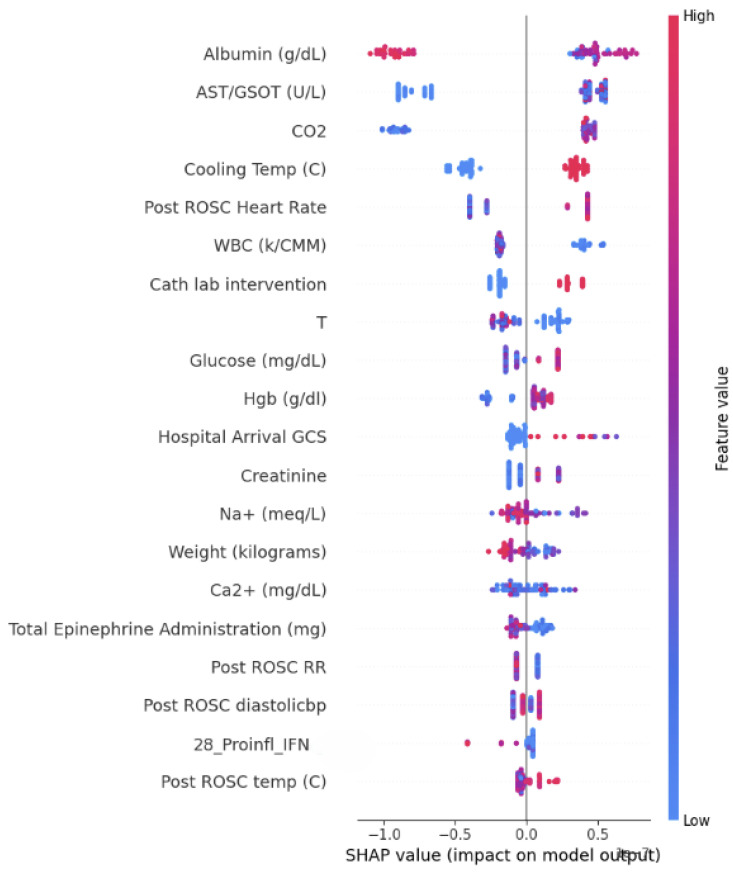
SHAP value impact for the model 1 HOUR ALL-Model 5.

**Figure 5:**
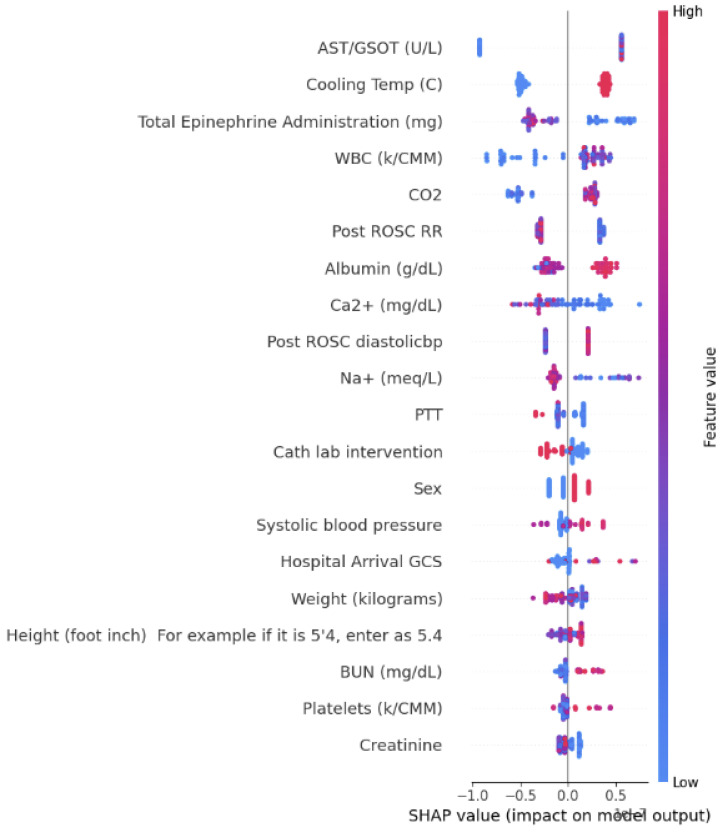
SHAP value impact for the model which includes the base model and biomarkers at 1 h, 6 h, and 24 h-Model 18.

**Figure 6:**
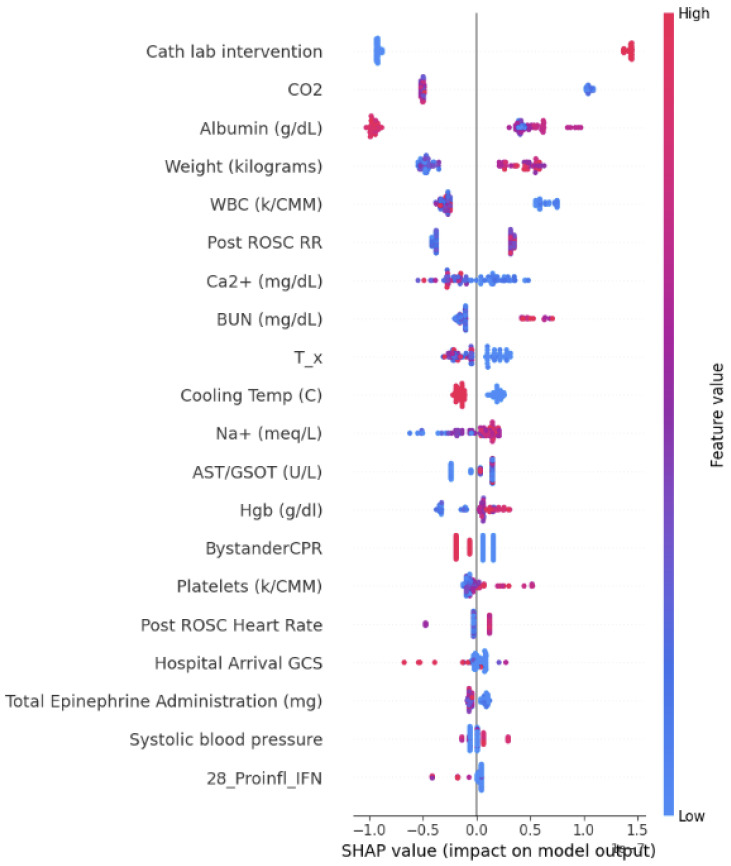
SHAP value impact for the model which includes 1 h and 6 h all variables and the biomarkers only at 24 h-Model 27.

The findings of the SHAP analysis are presented in Figure 4, Figure 5 and Figure 6. In these figures, the correlations are visually represented through color coding. A red color indicates a positive correlation between a feature and the outcome, while blue signifies a negative correlation with the outcome prediction.

## 4. Discussion

### 4.1. Summary of the Model Compilation

There have been many efforts intended to predict OHCA neurological outcomes. In this pilot study, utilizing NNs, we developed a base model and proposed several other combination models including commonly available and novel variables for 1 h, 6 h and 24 h. Existing studies have not explored the novel research variables included in this study, such as proinflammatory, chemokine, cytokine, angiogenesis, and vascular injury biomarkers, ocular ultrasound, and sex steroid hormones. To our knowledge, this is the first study to combine all typically available post-ROSC data points with novel research variables into a NN model to evaluate for the ideal combination of variables that can aid in neurological outcome prediction. Interestingly, the model with the best AUC had eight out of the top 10 variables that are easily available data elements for every post ROSC patient. Only two novel biomarkers, TARC and interferon gamma were features of high importance that are not typically available. This pilot data analysis using a NN methodology has provided insight into commonly available variables within the first hour after resuscitation from cardiac arrest and their contribution towards a high AUC score, indicating a good level of discrimination for predicting good CPC versus poor CPC outcome.

Amongst some of the successful prediction models for OHCA include the work led by Joon-myoung Kwon, who aimed to develop and validate a deep-learning-based out-of-hospital cardiac arrest prognostic system (DCAPS) for predicting neurologic recovery and survival to discharge. The study used patients from the Korea OHCA registry who experienced return of spontaneous circulation (ROSC) after OHCA. The Area Under the Receiver Operating Characteristic Curve (AUROC) of DCAPS was 0.953 with a 95% confidence interval 95% CI: [0.952–0.954], outperforming commonly used machine learning models such as logistics regression and support vector machine (SVM) [20]. Furthermore, initially, we tested commonly used machine learning models, including SVM, Random Forest (RF), and XGBoost. These models were excluded from the study since their performance was very low compared to the NN models used in this study.

Unique to our dataset are novel sets of variables including sex hormones, plasma protein biomarkers assessed at different time points post-ROSC, and point-of-care ultrasound ocular measurements. These exploratory variables were included in our model development to investigate additional hospital-level data points that may better refine outcome prediction. Our previous work has alluded to sex differences in outcomes as well as the role of specific sex hormones in association to survival outcomes. While NN approaches have been used to predict cardiac arrests, and ROSC, there has been limited literature on NN approaches to predict good neurologic outcome in OHCA patients. Such models could eventually improve clinical decision making at the patient-specific level with precise tailored interventions.

Our analysis has shown that the best performing model was model 5 with AUC of 0.946 in the training set and an AUC of 0.924 in the test set being quite similar, indicating a strong model for the outcome prediction of CPC. Furthermore, the variables of importance for model 5, identified by random forest variable importance analysis, illustrate that 8 out of the top ten variables are common, readily available hospital level variables and only 2 are novel variables with significant contributing power. The top 10 important variables for model 5, summarized in Figure 3, contribute to 60% of the prediction power of the CPC score. As a result, this pilot analysis provides insight that there is a large amount of data that is typically already collected in a cardiac arrest that may help ascertain neurological outcome prediction even at the first hour of data collection.

A second observation that can be made from our results and supports current research is that clinical variables such as post ROSC heart rate, hospital arrival GCS, and WBC (k/CMM) may have a major association in the outcome of OHCA patients. These common variables that are collected in the first hour are time sensitive and their values can be critical decision variables that provide clinicians with insight into the possible trajectory of the patient towards a binary neurological outcome. Individually, each of these common variables does not carry the level of importance, but when collectively placed in a model, these variables have surfaced as critical features for OHCA outcome prediction. Stratifying patients with the data variables already typically collected can help tailor a patient’s care plan. These variables deserve further attention using larger data sets to explore their significance.

Additionally, our preliminary NN models have also shown the added value of the research variables as important for developing more robust predictive models of OHCA neurological outcomes of CPC score. The second best model (model 18) comprises of the base model at 1, 6, and 24 h, and novel biomarkers at 1, 6, and 24 h, achieving an AUC score of 0.936. For all three time points, biomarkers have surfaced as an important predictor of CPC score. Our modeling has shown that inclusion of novel research variables results in higher AUC scores. Specifically, biomarkers have particularly contributed to the models in the later time points of 6 h and 24 h. To date, it is an uncommon phenomena for blood sample collection after cardiac arrest research purposes and paucity of literature on the biomarker trajectories after ROSC. As a result, there continue to be large knowledge gaps on the post-ROSC biomarker trajectories. Our pilot work highlights that further research with biomarker analysis of post-ROSC patients in a longitudinal manner could contribute meaningfully to OHCA outcome prediction.

The random forest based analysis for variables importance and the SHAP analysis indicate that interferon gamma (IFN) is a biomarker that has a high contribution to the CPC score prediction, as it appears important in many of the better predictive models. IFN is a pro-inflammatory cytokine, produced by lymphocytes and a potent activator of macrophages. Its role in cardiac arrest is unclear and deserves particular attention. A second biomarker that should be particularly considered is Thymus and activation regulated chemokine(TARC) as it is in the top 10 features for hour 1 (Figure 3). TARC is another chemokine that is involved in the inflammatory cascade and has been shown to be an early prognostic biomarker of severity of inflammation [21], but once again, its role within cardiac arrest is unclear and requires further exploration.

Our pilot analysis is at most hypothesis generating has provided a novel perspective into the untapped potential of already existing clinical data variables that can potentially guide medical decision making for OHCA. It also provides justification for the development of a OHCA data collection tool that incorporates in-hospital variables. The Institute of Medicine (IOM) has long called for a national cardiac arrest registry with incorporation of data elements from pre-hospital and in-hospital. Our pilot study provides further proof of the value added with the collection of these commonly available variables at the hospital level so that complex methodologies such as NN can be applied to this complex medical problem.

### 4.2. Study Limitations

The study focused on patients who achieved ROSC and survived to the ED, therefore, this study is not representative of all OHCA cases. As a result, the sample may introduce a selection bias that could limit the generalizability and applicability of the research findings.

Furthermore, the sample size of this study, limited to 107 OHCA patients, constrains the generalizability of the findings. A small cohort like this reduces the statistical power and robustness of the conclusions. Also, the evaluation provided for the NN model might not be reasonable due to the small size of the data set. Additionally, the limited data set increases the risk of model overfitting, which can result in spurious associations and reduced external validity. Furthermore, missing data from clinical variables and exploratory novel variables led to variations in sample size from one model to another. For instance, the biomarker data was available for only 33 patients from a cohort of 107. We also did not create base models for hours 6 and 24 since clinicians would not use data points in isolation, but rather use all the previous data points that are available to make a decision. Hence, isolated base models at 6 and 24 h without utilizing the prior data did not seem to be a practical approach.

Another constraint is the limited interpretability of NN models, despite their predictive capabilities. The features of importance and SHAP values are only applicable to this particular data set. Any alteration in the set of variables may affect the contribution of the variable. Hence, the complexity of these models currently makes it difficult to translate into clinical practice and incorporate into medical decision making without access to larger data sets and replication of these models.

Although this research is in its early stages, this study contributes to our current understanding of cardiac arrest prediction models by exploring the value of currently available variables at the hospital level. In addition, very rarely are blood samples collected for research purposes for post-ROSC patients; our pilot study showcases the potential added value of novel variables that have never been explored in CPC prediction models.

## 5. Conclusions

Cardiac arrest is the most complex, multi-organ medical malady that afflicts our society in vast numbers. With the application of NN, we aimed to gain new insight into features and variables that are critical for cardiac arrest outcome prediction. Predicting CPC scores is crucial for efficient resource allocation as well as refining care at the individual patient level. By identifying individuals who may benefit from specific therapies, we can better direct resources. This knowledge also aids in informed discussions with families post-cardiac arrest, ensuring support is focused where it is most needed.

Our pilot study explored 27 models with multiple combinations of 121 total variables available at the hospital in conjunction with novel research variables to predict neurological outcome. We discovered that commonly used clinical variables at the hospital level in a NN model have strong prediction capability of the neurological outcome after OHCA. We also uncovered that the combination of plasma biomarkers, optic nerve ultrasound measurements, and sex hormones contributed to the best model prediction. Future studies with larger data sets will require a more granular analysis of how much contribution each of these subsets of novel variables actual contributes towards neurological outcome prediction.

## Figures and Tables

**Table 1:** Comparison of Variables: CPC score, Age, Sex, Race, Bystander CPR, and Initial Arrest Rhythm.

Characteristics	Training Cohort (N = 74)	Testing Cohort (N = 33)	*p*-Value
**Outcome Variable N (%)**			
CPC score 1–2	15 (20.3)	6 (18.2)	0.804
CPC score 3–5	59 (79.7)	27 (81.8)	0.804
**Demographics**			
Age Mean (Std)	62 (53.0)	58 (52.0)	0.838
*Sex N (%)*			
Female	37 (50.0)	11 (33.3)	0.178
Male	37 (50.0)	22 (66.7)	
*Race N (%)*			
White	20 (27.0)	7 (21.2)	0.527
Other	54 (73.0)	26 (78.8)	
**Clinical N (%)**			
*Bystander CPR*			
Yes	32 (43.3)	11 (33.3)	0.339
No	42 (56.8)	22 (66.7)	
*Initial Arrest Rhythm*			
Asystole/PEA	51 (68.9)	23 (69.7)	0.937
Ventricular Fibrillation/Ventricular Tachycardia	23 (31.1)	10 (30.3)	

**Table 2:** Test results of best performing models, model 5, model 18 and model 27.

Model	AUC Test Set Score
Model 5	0.924
Model 18	0.898
Model 27	0.886

## Data Availability

The datasets generated and/or analyzed during the current study are not publicly available due to medical patients requiring consent, but are available from the corresponding author on reasonable request.

## Abbreviations

The following abbreviations are used in this manuscript:

OHCA	Out-of-hospital cardiac arrest
NN	Neural Network
DREAM	Decay Replay Mining

HF	Heart Failure
IFN	Interferon Gamma
TARC	Thymus and Activation-Regulated Chemokine
ROSC	Return of Spontaneous Circulation
CPC score	Cerebral Performance Category score is a measure of neurologic outcome
